# Coexistence of Charcot-Marie-Tooth 1A and nondystrophic myotonia due to *PMP22* duplication and *SCN4A* pathogenic variants: a case report

**DOI:** 10.1186/s12883-021-02538-5

**Published:** 2022-01-07

**Authors:** Haitian Nan, Yunqing Wu, Shilei Cui, Houliang Sun, Jiawei Wang, Ying Li, Lingchao Meng, Takamura Nagasaka, Liyong Wu

**Affiliations:** 1 Department of Neurology, Xuanwu Hospital, Capital Medical University, Beijing, China; 2 Department of Neurology, Beijing Tongren Hospital, Capital Medical University, Beijing, China; 3 Department of Neurology, Peking University First Hospital, Beijing, China; 4 Department of Neurology, University of Yamanashi, 1110 Shimokato, Chuo-city, Yamanashi, 409-3898 Japan

**Keywords:** Charcot-Marie-Tooth disease, Nondystrophic myotonia, *PMP22*, *SCN4A*, Case report

## Abstract

**Background:**

Charcot-Marie-Tooth disease (CMT) is a genetically heterogeneous hereditary neuropathy, and CMT1A is the most common form; it is caused by a duplication of the peripheral myelin protein 22 (*PMP22*) gene. Mutations in the transient sodium channel Nav1.4 alpha subunit (*SCN4A*) gene underlie a diverse group of dominantly inherited nondystrophic myotonias that run the spectrum from subclinical myopathy to severe muscle stiffness, disabling weakness, or frank episodes of paralysis.

**Case presentation:**

We describe a Chinese family affected by both CMT1A and myotonia with concomitant alterations in both the *PMP22* and *SCN4A* genes. In this family, the affected proband inherited the disease from his father in an autosomal dominant manner. Genetic analysis confirmed duplication of the *PMP22* gene and a missense c.3917G > C (p. Gly1306Ala) mutation in *SCN4A* in both the proband and his father. The clinical phenotype in the proband showed the combined involvement of skeletal muscle and peripheral nerves. Electromyography showed myopathic changes, including myotonic discharges. MRI revealed the concurrence of neurogenic and myogenic changes in the lower leg muscles. Sural nerve biopsies revealed a chronic demyelinating and remyelinating process with onion bulb formations in the proband. The proband’s father presented with confirmed subclinical myopathy, very mild distal atrophy and proximal hypertrophy of the lower leg muscles, *pes cavus*, and areflexia.

**Conclusion:**

This study reports the coexistence of *PMP22* duplication and *SCN4A* mutation. The presenting features in this family suggested that both neuropathy and myopathy were inherited in an autosomal dominant manner. The proband had a typical phenotype of sodium channel myotonia (SCM) and CMT1A. However, his father with the same mutations presented a much milder clinical phenotype. Our study might expand the genetic and phenotypic spectra of neuromuscular disorders with concomitant mutations.

**Supplementary Information:**

The online version contains supplementary material available at 10.1186/s12883-021-02538-5.

## Background

Charcot-Marie-Tooth disease (CMT) comprises the most common group of disorders of the peripheral nervous system and is clinically and genetically heterogeneous. The most common CMT type is CMT1A, which is due to a dominantly inherited 1.5 megabase duplication on the short arm of chromosome 17 at locus p11.2 [1]. This duplicated segment contains the *PMP22* gene that encodes peripheral myelin protein 22.

Nondystrophic myotonias (NDMs) are a group of hereditary muscle diseases characterized by myotonia, muscle stiffness, and a nondystrophic phenotype, which are caused predominantly by mutations in *CLCN1* or *SCN4A* [2]. *SCN4A* myotonias are clinically classified into three subgroups: sodium channel myotonia (SCM), paramyotonia congenita (PMC), and hyperkalemic periodic paralysis (Hyper PP) [3].

Because the estimated prevalence of SCM is approximately 0.06 per 100,000 population [4] and the calculated prevalence of CMT1A is in the range of 26:100,000 to 8:100,000 [5], the theoretical chance of inheriting both diseases is extremely low. Herein, we describe a Chinese family with a 1.5 megabase duplication including *PMP22* and a heterozygous mutation G1306A in *SCN4A*. The proband had a typical phenotype of both SCM and CMT1A. However, his father with the same mutations presented a much milder clinical phenotype.

## Case presentation

### Clinical study

**Fig. 1:**
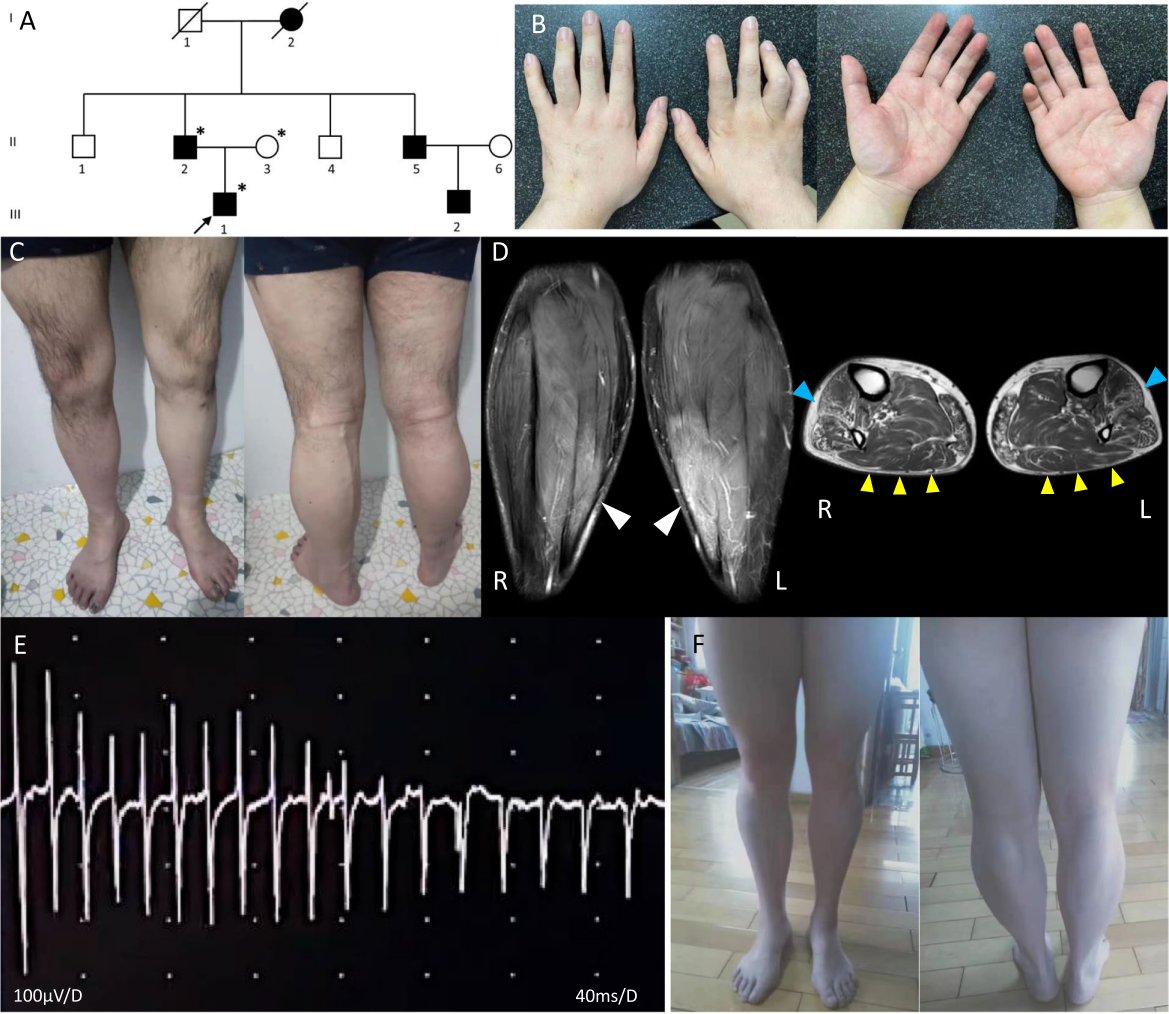
**A** Pedigree of the present family. The proband is indicated (arrow). Individuals evaluated both clinically and genetically in this study are denoted by asterisks. **B** Serial photographs of the proband taken at age 29. The proband presented hand deformities characterized by curvature of the fingers. There was mild atrophy of the distal muscles of the hands, particularly of the thenar eminence. **C** Appearance of the legs of the proband. There was mild quadriceps wasting and simultaneous mild calf hypertrophy. **D** Fat-saturated MRI of the lower legs in the coronal plane and T2-weighted image in the axial plane of the lower third of the calves of the proband. High-intensity regions manifesting as neurogenic changes can be easily observed in the bilateral triceps surae muscles, with proximal dominant hypertrophy in the coronal plane (white arrowhead). There was moderate fatty atrophy involving bilateral tibialis anterior muscles in the axial plane (blue arrowhead), which predominated on the right side. Conversely, there was very slight hypertrophy in the posterior compartment muscles (yellow arrowhead), including the soleus, gastrocnemius, and tibialis posterior. R and L indicate right and left. **E** Electrophysiologic findings in the proband recorded from the left extensor digitorum communis. The insertion activity of needle electromyography at rest shows myotonic discharges (timebase set to 40 ms/division). **F** Appearance of the legs of the proband’s father at age 57. *Pes cavus* is pronounced. Mild proximal hypertrophy and distal atrophy of bilateral lower leg muscles were observed

The pedigree is shown in Fig. 1 A. The proband (Fig. 1 A, III-1), a 29-year-old male, was the only child of unrelated parents. His development was normal. As a child, he kept up with his peers physically. However, he displayed high-arched feet since adolescence. He began experiencing muscle stiffness in both hands and legs from his late teens, mostly in the mornings. Stiffness could also appear after a long exercise. Progressive leg weakness with drop feet and a steppage gait began insidiously during his twenties. At 28 years of age, his leg weakness worsened, and he developed numbness in the distal upper and lower extremities. At age 29, distal weakness of the hands affecting the dorsal interossei with reduced abduction in the metacarpophalangeal joints and weakness in thumb abduction occurred, and he presented hand deformities characterized by curvature of the fingers (Fig. 1 B). The patient reported hypoesthesia of fingers and toes with poor finger control, and he had difficulties buttoning up his clothes or using chopsticks. Myotonia could be induced by hand grip and was relieved by tapping or massage for 4–5 s. Symptoms were not exacerbated by cold exposure. There was no typical warm-up phenomenon, and he never experienced myalgia or paralytic attacks. Currently, he is still ambulatory with crutches.

Neurological examination revealed a steppage gait, drop feet, *pes cavus* deformity, hammertoes, claw hand, grip myotonia, and symmetric distal upper and lower extremity weakness. The bilateral proximal muscle strength of the upper extremities and lower extremities was normal. The strength in distal muscles of the upper extremities, including the wrist flexors and extensors, was 3/5 on the medical research council scale (MRC). The strength in the intrinsic hand muscles, including the finger flexors, the first dorsal interosseous, the abductor digiti minimi, and the abductor pollicis brevis, was MRC grade 2/5. The muscle strength levels of the ankle and toe extensors were MRC grades 3/5 and 2/5, respectively, while ankle dorsiflexion was completely restricted on both sides. There was mild atrophy of the distal muscles of the hands, particularly of the thenar eminence (Fig. 1 B). Subtle wasting of the thigh musculature was noted, whereas very mild calf hypertrophy was also identified (Fig. 1 C). He had difficulty with tandem gait and had decreased sensitivity to vibration and pain in a stocking-glove distribution involving the upper limbs to the level of the elbows and the lower limbs to the mid-calf. Deep tendon reflexes were absent, and plantar reflexes were flexor. There were no abnormalities in the cranial nerves or his coordination. Stork legs, scoliosis, lid lag, dysphagia, dysarthria, myokymia, fasciculations, joint malformations, or pyramidal tract signs were not present.

His creatine kinase (CK) levels were 224 U/L and 386 U/L on repeated measurements (reference range, 25–240 U/L). The liver function tests, albumin, serum potassium levels, thyroid function, and autoantibody levels (including anti-Jo1 and anti-acetylcholine receptor) were within normal ranges. His brain and spinal magnetic resonance imaging (MRI) results were normal. His echocardiogram was normal without signs of hypertrophy and with normal relaxation time. Muscle MRI of the proband showed the concurrence of both fatty degeneration and very mild hypertrophy in the lower leg muscles (Fig. 1 D).

Needle electromyography (EMG) was performed in the right abductor policis brevis, the left extensor digitorum muscle, the left biceps muscle, the left gastrocnemius muscle, the right vastus medialis, and the right tibialis anterior muscle. All muscles demonstrated typical runs of myotonic discharges (Fig. 1 E). Unfortunately, the patient rejected exerting strength with pain, so we could only record insertion activity. We observed some motor unit action potentials (MUAPs), with long duration relative to modest amplitude (amplitude 0.2–0.9 μV, duration 3-7 ms) with decreased interference, in the extensor digitorum communis, which appeared in a short time before myotonic discharge (an additional movie file shows this in more detail [see Additional file 1]). MUPs may reveal the coexistence of a muscle disorder as well as neuropathy. Motor nerve conduction velocities were markedly decreased (median nerve, 15.5 m/s; ulnar nerve, 17 m/s; tibial nerve, 13.5 m/s; peroneal nerve, 16.8 m/s). Distal motor latencies were prolonged (median nerve, 15.4 ms; ulnar nerve, 12 ms; tibial nerve, 12.2 ms; peroneal nerve, 8.16 ms), and compound muscle action potential (CMAP) amplitudes were markedly decreased (median nerve: 0.43 mV, ulnar nerve: 0.21 mV, tibial nerve: 0.074 mV, peroneal nerve: 0.11 mV). Sensory nerve action potentials (SNAPs) were not elicited in the upper or lower extremities. Nerve conduction study results were consistent with motor and sensory demyelinating neuropathy (Table 1). Decreased CMAP amplitude could be a reflection of the reduced muscle fiber volume due to severe fiber loss.

**Table 1:** Electrophysiologic studies of the proband reported in this study

	Proband	Normal range
Median nerves		
DML (ms)	15.4	< 4.4
MCV (m/s)	15.5	> 49
Proximal CMAP (mV)	0.43	> 4
Ulnar nerves		
DML (ms)	12	< 3.3
MCV (m/s)	17.0	> 49
Proximal CMAP (mV)	0.21	> 6
SCV (m/s)	25.3	> 47
SNAP (_μ_v)	–	> 3
DML (ms)	4.75	
Peroneal nerves		
DML (ms)	8.16	< 5.8
MCV (m/s)	16.8	> 41
Proximal CMAP (mV)	0.11	> 4
Tibial nerves		
DML (ms)	12.2	< 5.8
MCV (m/s)	13.5	> 41
Proximal CMAP (mV)	0.074	> 4

*DML* distal motor latency, *MCV* motor conduction velocity, *CMAP* compound muscle action potential, *SCV* sensory conduction velocity, *SNAP* sensory nerve action potential

The proband’s father (Fig. 1 A, II-2), a 57-year-old male, presented *pes cavus* feet and no other related manifestations. His deep tendon reflexes were absent. The remainder of the neurological examination was normal, including sensory and manual muscle testing. He showed very mild distal atrophy and proximal hypertrophy of the lower leg muscles (Fig. 1 F). Despite not having symptoms related to myotonia, myotonic discharges were confirmed in the left biceps muscle on needle EMG examination (see Additional file 2). Unfortunately, a nerve conduction study of the proband’s father was not performed, and we could not obtain more information.

**Fig. 2:**
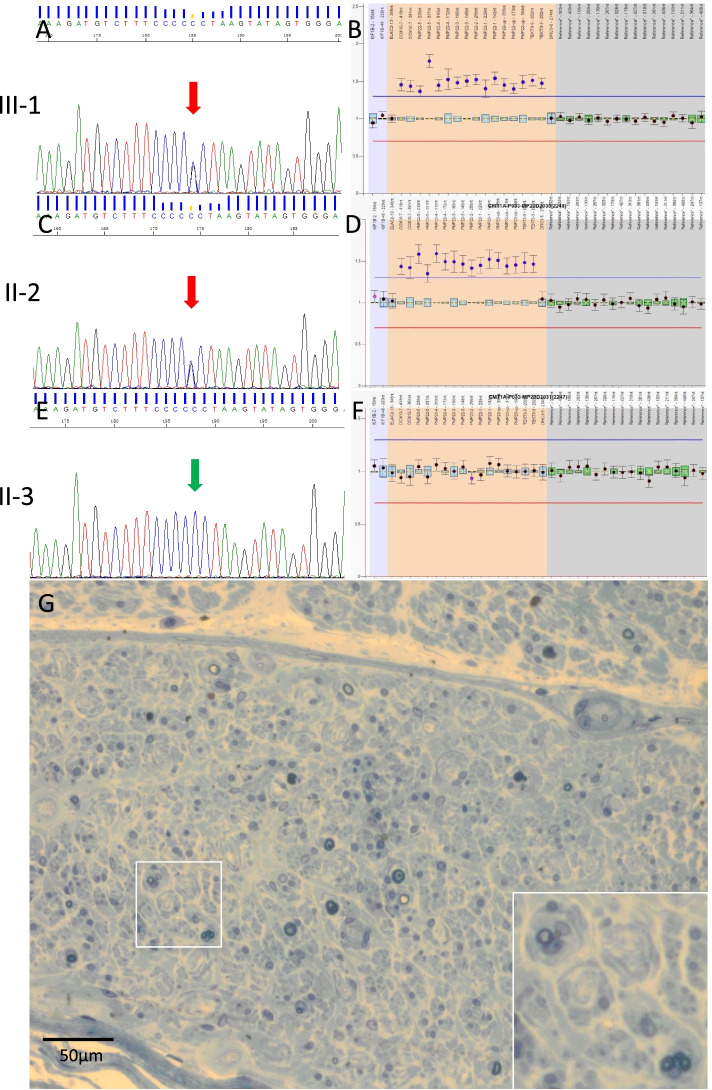
**A** Sanger sequencing revealed that the c.3917G > C mutation in *SCN4A* was heterozygous in the proband. **B** The results of MLPA study in the proband. A heterozygous 1.5-Mb duplication in 17p11.2-p12, including the coding regions of the *COX10*, *PMP22*, and *TEKT3* genes, was found. Data were analyzed by Coffalyser software. The cutoff value for duplication was > 1.2. **C** Sanger sequencing revealed that the c.3917G > C mutation in *SCN4A* was heterozygous in the proband’s father. **D** The results of MLPA study in the proband’s father. A heterozygous 1.5-Mb duplication in 17p11.2-p12, including the coding regions of the *COX10*, *PMP22*, and *TEKT3* genes, was found. **E** The c.3917G > C mutation in *SCN4A* was not detected in the proband’s mother. **F** The results of MLPA study in the proband’s mother. No duplication in the coding regions of the *COX10*, *PMP22*, or *TEKT3* genes was found. **G** The sural nerve biopsy of the proband was examined by light microscopy. Toluidine blue staining revealed severe loss of myelinated fibers and regenerating clusters of myelinated fibers, with frequent onion bulb formations

The proband’s deceased paternal grandmother (Fig. 1 A, I-2), his paternal uncle (Fig. 1 A, II-5), and his paternal uncle’s son (Fig. 1 A, III-2) also had *pes cavus*, but they have not been reviewed at our center. They did not report any neurological symptoms. No other family members are known to be affected in this pedigree. The proband’s mother (Fig. 1 A, II-3) did not show any neurological abnormalities.

### Genetic study

We carried out whole-exome sequencing of genomic DNA from the proband. Genomic DNA was isolated from peripheral blood leukocytes using standard methods. Exome capture was performed with a SureSelect Human All Exon V6 + UTR (89 Mb) Kit (Agilent Technologies, Santa Clara, CA, USA). Paired-end sequencing was carried out on a HiSeq2500 (Illumina, San Diego, CA, USA) using a HiSeq SBS Kit V4 (Illumina), which generated 100-bp reads. The reference databases utilized included hg19 (GRCh37) (http://genome.ucsc.edu), HGMD (https://portal.biobase-international.com), GnomAD (http://gnomad.broadinstitute.org), and dbSNP (https://www.ncbi.nlm.nih.gov/snp/). We examined variants of genes known to be responsible for neuromuscular diseases such as CMT or myotonia (https://neuromuscular.wustl.edu/). Through this analysis, we identified a c.3917G > C (p. Gly1306Ala) mutation in exon 22 of the *SCN4A* gene (NM_000334) and ruled out point mutations in other causative genes for neuromuscular diseases. We then carried out Sanger sequencing of genomic DNA from the proband (Fig. 1 A, III-1), the proband’s father (Fig. 1 A, II-2), and the proband’s mother (Fig. 1 A, II-3). We reconfirmed the c.3917G > C (p. Gly1306Ala) mutation in exon 22 of the *SCN4A* gene, which was in a heterozygous state in the proband (Fig. 2 A) and his father (Fig. 2 C). However, this mutation was not detected in the proband’s mother, who was without symptoms (Fig. 2 E).

Furthermore, we used our in-house script aimed to find copy number variants (CNVs) and genotype the copy numbers of duplicated genes by analyzing exome sequencing data. We detected a probable duplicated segment on the short arm of chromosome 17 at locus p11.2, which contains the *PMP22* gene in the proband. Multiplex ligation-dependent probe analysis (MLPA) was then used to verify the duplications in the *PMP22* gene. MLPA was performed using the MLPA kit (SALSA MLPA probemix P405-A1 CMT; MRC Holland, Amsterdam, Netherlands) according to the manufacturer’s protocol. Cutoff values for duplication and deletion were set as > 1.2 and < 0.7, respectively [6]. MLPA studies revealed the presence of a heterozygous 1.5 Mb duplication in 17p11.2-p12, including the coding regions of *PMP22* in the proband (Fig. 2 B) and the proband’s father (Fig. 2 D). However, this duplication in *PMP22* was negative in the proband’s mother (Fig. 2 F). We thus demonstrated that both the duplication in *PMP22* and the p.G1306A mutation in *SCN4A* in the proband were inherited from his father.

### Neuropathological study

Sural nerve biopsy was performed in the proband with both *SCN4A* and *PMP22* alterations. The samples were initially fixed in 2.5% glutaraldehyde, followed by 1% buffered osmium tetroxide, and then dehydrated in ascending grades of ethanol and embedded in Epon. Transverse semithin sections were stained with toluidine blue. Sural nerve biopsies revealed a severe loss of myelinated fibers. There was some axonal degeneration and regenerating clusters of myelinated fibers. Onion bulb formation was frequent (Fig. 2 G). Unfortunately, we failed to obtain muscle tissues during the biopsy due to severe fatty degeneration of the distal lower leg muscles.

## Discussion and conclusions

The occurrence of two genetically unlinked disorders in a patient or a pedigree is rare, but studies include descriptions of a boy with myotonia congenita (MC) and CMT1A [7], patients affected with both MC and *SCN4A*-myotonias [8–10], and a family with a mutation identified in *MPZ* in two patients with CMT and a mutation identified in *SCN4A* in 4 family members with Hyper PP [11]. To our knowledge, our report represents the first case of cosegregation of *SCN4A* myotonias and CMT1A.

The G1306A missense variant in the *SCN4A* gene has been reported multiple times in association with myotonia. Nav1.4-G1306 is localized in the III-IV intracellular loop of the voltage-gated sodium channel of skeletal muscle, which contains the fast inactivation particle. At this position, there are three observed genotypes to be distinguished, of which G1306V and G1306E often lead to more severe phenotypes [12], while G1306A mostly leads to the least severe, a mild form of SCM [13], characterized by nondystrophic, generalized myotonia with daily fluctuations and muscle stiffness after exercise without substantial cold sensitivity [14–18]. Muscle pain is not a frequent symptom [12], while CK values are frequently elevated in such patients, with average elevations of 2–3-fold, indicating mild myolysis [12]. In our proband, myotonia was also observed both clinically and electrophysiologically. He had handgrip myotonia, postexercise myotonia, and mild muscle hypertrophy. He also showed myotonic discharges on electromyography. His CK levels were either normal or mildly elevated. These findings suggest an SCM phenotype, thus supporting the pathogenic role of the *SCN4A* mutation. Moreover, muscle weakness and atrophy, sensory disturbance, and abnormal tendon reflexes were also detected, which phenotypically fit into CMT1A.

Mutations of the *PMP22* gene in combination with other genetic variants have been previously reported to cause a more severe phenotype than the one expected by *PMP22* mutation alone [19]. Interestingly, the proband’s father displayed a milder phenotype despite the presence of both *PMP22* and *SCN4A* variations. He was not aware of any neurologic or myogenic symptoms, but myotonia was observed in the electrophysiological examination. The clinical features of the proband and his father are summarized in Table 2. There is an anticipation phenomenon in CMT1A patients [20]. Moreover, the penetrance of *SCN4A* myotonias is high, particularly in males. In this family, apparent genetic anticipation of peripheral neuropathy was also observed. The CMT1A symptoms of muscle weakness, sensory disturbance, and steppage gait of the proband were absent in the proband’s father. However, the proband had a relatively mild myotonia phenotype, so genetic anticipation of myotonia could not be concluded in this family. However, we cannot predict whether this condition in our proband could lead to more severe clinical impairment with age. It is of interest that the proband and his father presented different clinical phenotypes even though they had the same mutations. There might be some unknown genetic etiology or environmental modifiers underlying the phenotypes that could not be identified with our present methods. Further functional study may be warranted to identify the underlying cellular and molecular mechanisms for this disease.

**Table 2:** Clinical features of the proband and his father with *PMP22* and *SCN4A* mutations

Patient		Proband	Proband’s father
*SCN4A* gene studies		c.3917G > C p. Gly1306Ala	c.3917G > C p. Gly1306Ala
*PMP22* gene studies		Duplication in *PMP22*	Duplication in *PMP22*
Sex		Male	Male
Age (yrs)		29	57
Age at Onset		Adolescence	Unknown
First symptom at onset		High-arched feet	High-arched feet
Muscular Stiffness		Yes, mostly in the mornings or after long exercises	No
Muscle pain		No	No
Muscle weakness	Upper limb	Wrist muscles 3/5, intrinsic hand muscles 2/5	No
	Lower limb	Ankle and toe extensors < 3/5, ankle dorsiflexion restricted	No
Muscle atrophy		Mild atrophy of the distal muscles of the hands, subtle wasting of thigh musculature	Very mild distal atrophy of the lower leg muscles
Muscle hypertrophy		Mild calves hypertrophy	Very mild proximal hypertrophy of the lower leg muscles
Warm up phenomenon		No	Not Applicable
Handgrip myotonia		Yes	No
Myotonia worsened by cold		No	Not Applicable
Myotonia distribution		Hands, arms, legs	Not Applicable
Electromyography		Positive for generalized typical myotonic discharges	Myotonic discharges were confirmed in the left biceps muscle
CK(U/L)		224 U/L, 386 U/L	Not examined
*Pes cavus*		Yes	Yes
Sensory loss		A stocking-glove distribution involving the upper limbs to the level of the elbows and the lower limbs to the mid-calf	No
Deep tendon reflexes		Absent	Absent
Plantar response		Flexor	Flexor
Sural nerve biopsy		Severe demyelinating neuropathy	Not examined
CMTNS		Severe (26)	Mild
Recurrent flaccid paralysis		No	No
Scoliosis		No	No

*Abbreviations*: *CMTNS* CMT neuropathy score

At first, pure myotonia went unnoticed in the proband because of a preponderance of the more severe condition of peripheral neuropathy. It was not until the *SCN4A* mutation was identified by WES analysis that we stepped back and recollected the medical data of the proband. Clinicians should consider routinely performing comprehensive genetic tests for patients with atypical symptoms, even in the case that one pathological mutation has already been identified.

In conclusion, we present a family with a combination of mutations in *PMP22* and *SCN4A,* leading to the coexistence of two autosomal dominantly inherited disorders: CMT1A and SCM. The clinical phenotype in the proband showed the combined involvement of skeletal muscle and peripheral nerves. His father with the same mutations presented a much milder phenotype. This rare genetic coincidence might expand the genetic and phenotypic spectra of neuromuscular disorders with concomitant mutations.

## Supplementary Information

**Additional file 1.** EMG of the proband recorded from the left extensor digitorum communis showed complex repetitive discharges and myotonic discharges.

**Additional file 2.** EMG of the proband’s father recorded from the left biceps muscle showed myotonic discharges.

**Additional file 3.**

## Data Availability

All data generated or analyzed during this study are included in this published article.

## Abbreviations

CMT: Charcot-Marie-Tooth disease
SCM: Sodium channel myotonia
NDM: Nondystrophic myotonia
PMC: Paramyotonia congenita
Hyper PP: Hyperkalemic periodic paralysis
MRC: Medical research council scale
MRI: Magnetic resonance imaging
EMG: Electromyography
CMAP: Compound muscle action potential
SNAP: Sensory nerve action potentials
CNV: Copy number variant
MLPA: Multiplex ligation-dependent probe analysis
PCR: Polymerase chain reaction
MC: Myotonia congenita
CK: Creatine kinase
